# Quantum controlled-phase-flip gate between a flying optical photon and a Rydberg atomic ensemble

**DOI:** 10.1038/srep10005

**Published:** 2015-05-12

**Authors:** Y. M. Hao, G. W. Lin, Keyu Xia, X. M. Lin, Y. P. Niu, S. Q. Gong

**Affiliations:** 1 Department of Physics, East China University of Science and Technology, Shanghai 200237, China; 2 ARC Centre for Engineered Quantum Systems, Department of Physics and Astronomy, Macquarie University, NSW 2109, Australia; 3 College of Physics and Energy, Fujian Normal University, Fuzhou 350108, China

## Abstract

Quantum controlled-phase-flip (CPF) gate between a flying photon qubit and a stationary atomic qubit could allow the linking of distant computational nodes in a quantum network. Here we present a scheme to realize quantum CPF gate between a flying optical photon and an atomic ensemble based on cavity input-output process and Rydberg blockade. When a flying single-photon pulse is reflected off the cavity containing a Rydberg atomic ensemble, the dark resonance and Rydberg blockade induce a conditional phase shift 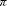 for the photon pulse, thus we can achieve the CPF gate between the photon and the atomic ensemble. Assisted by Rydberg blockade interaction, our scheme works in the *N*-atoms strong-coupling regime and significantly relaxes the requirement of strong coupling of single atom to photon in the optical cavity.

Quantum networks, composed of quantum channels and local nodes, provide opportunities and challenges across a range of intellectual and technical frontiers, including quantum computation, communication and metrology^1^. In a quantum network, photons are ideal flying qubits for carrying quantum information between the local nodes, while atoms are good candidates for stationary qubits which can be locally stored and manipulated in local nodes^2^,^3^,^4^. Therefore, quantum controlled-phase-flip (CPF) gate between a flying photon qubit and a stationary atomic qubit is a key component of the scalable quantum computational network^5^. Based on the cavity input-output process, Duan and Kimble^6^ proposed an interesting scheme to realize the quantum CPF gate between a flying photon and a single atom for scalable photonic quantum computation. By following this seminal scheme^6^, many theoretical schemes have been proposed for scalable quantum computation^7^,^8^,^9^,^10^,^11^,^12^ and long-distance quantum communication^13^,^14^,^15^ with the strong coupling of single atom to photon in an optical cavity. Very recently, the experiments successfully demonstrated this quantum CPF gate mechanism for nondestructive detection of an optical photon^16^, generation of entangled states^17^, and nanophotonic quantum phase switch^18^. All these schemes^6^,^7^,^8^,^9^,^10^,^11^,^12^,^13^,^14^,^15^,^16^,^17^,^18^ for photon-atom quantum gate explore strong coupling of single atom to photon with the high single-atom cooperativity 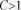, which requires stringent experimental conditions and thus greatly restricts their applications in the quantum network.

In this paper, based on the cavity input-output process and Rydberg blockade^19^,^20^, we present a scheme to realize the quantum CPF gate between a flying optical photon and an atomic ensemble. In our scheme, a Rydberg atomic ensemble is trapped in a single-sided optical cavity. When a flying single-photon pulse is reflected off the cavity, if there is no Rydberg excitation, the dark resonance induces a phase shift 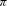 for the photon pulse, whereas if there is one Rydberg excitation, the Rydberg blockade interaction will move the atomic system out of the dark state and the photon pulse will bounce back with no phase shift. Thus we can achieve the CPF gate between the photon and the atomic ensemble. Assisted by Rydberg blockade interaction, our scheme works in the *N*-atoms strong-coupling regime, i.e., the collective cooperativity 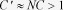. With a large number of atoms (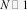), our scheme can work in the single-atom weak-coupling regime, i.e., 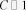, which significantly relaxes the requirement of the optical cavity for realization of the quantum CPF gate.

## Results

**Figure 1:**
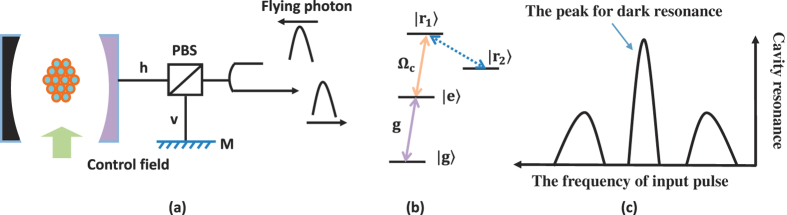
(Color online) (**a**) Schematic setup to realize the quantum controlled-phase-flip (CPF) gate between a flying photon qubit and a stationary atomic qubit. With a polarization beam splitter (PBS), the h polarization component of the single-photon pulse is reflected by the cavity, while the v polarization component is reflected via the mirror M. (**b**) The relevant level structure and transitions of the Rydberg atomic ensemble trapped in the cavity. (**c**) Schematic drawing for three resonant peak with three-level cavity-EIT system. The central peak results from dark resonance^27^.

As illustrated in Fig. 1(a), the basic building block of our scheme is an ensemble of 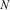 Rydberg atoms trapped inside a single-sided optical cavity, which reflects off a flying single-photon pulse. The relevant atomic level structure and transitions are shown in Fig. 1(b). Each atom has a stable ground state 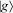, an excited state 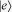, and two Rydberg states 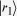 and 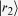. The atomic transition 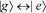 is resonantly coupled to the cavity mode 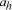 with horizontal (h) polarization, while a classical control field with Rabi frequency 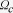 drives the transition 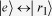. Thus they form the standard three-level electromagnetically induced transparency (EIT) configuration^21^,^22^,^23^, in which the coherent processes are described by interaction Hamiltonian 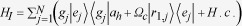. Assuming that almost all atoms are in the reservoir state 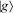 at all times, we can rewrite the Hamiltonian 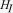 in terms of the collective states as


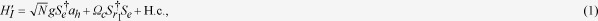


where 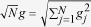
23 is the effective atom-cavity coupling strength, which is collectively enhanced due to the many-atom interference effect^24^. The collective operator is defined by 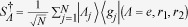. We consider the blockade interaction between Rydberg states 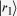 and 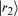 with the Hamiltonian


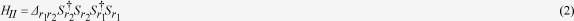


in terms of the collective states, here 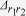 is additional energy shift when two atoms are excited to Rydberg states 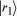 and 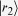, respectively^20^. Then the total Hamiltonian for the combined system (atoms + cavity mode + free space) has the following form in the rotating frame^25^


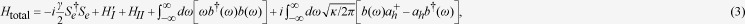


where 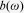 denotes the annihilation operator of free-space modes with the commutation relation 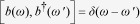, 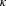 is the decay rate of the cavity mode, and 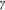 is the spontaneous emission rate of the atomic excited state, and the spontaneous emissions of Rydberg states are neglected due to their long coherence time.

In this paper, two initial states for atomic qubit are considered: i) state 
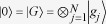
, i.e., all atoms are in the reservoir state; ii) single Rydberg excited state 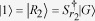. When the single photon is reflected off the cavity containing the atoms in state 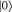 or 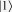, the whole state of the system at arbitrary time can be described by 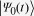 or 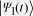, with


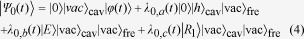


and


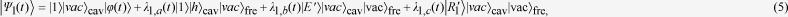


where 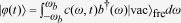 is the single-photon pulse with 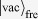 being the vacuum state of the free-space modes, 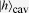 (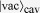) represents the single-photon Fock state (the vacuum state) of cavity mode, 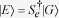 and 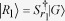 (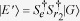 and 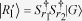) are one-atom (two-atom) excitation states of the atomic ensemble. According to the Schrödinger equation 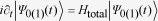, we have


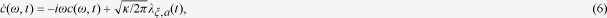



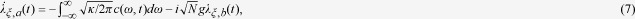



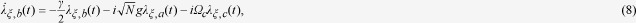



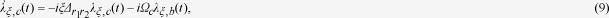


where 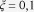 denotes that the initial state of atoms is 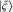.

Equations (6, 7, 8, 9) determine the evolution of the combined system, and can be solved without further approximation through numerical simulation. However, we can attack this problem analytically with some rough approximations to reveal the underlying physics. Then we find that the cavity output 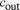 is connected with the input 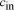 by (see Methods)


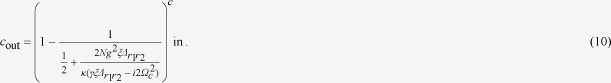


When 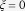, i.e., the atoms are initially in state 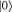, this expression simplifies to


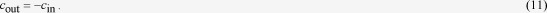


When 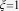, i.e., the atoms are initially in state 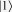, if condition


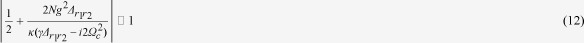


is satisfied, we have


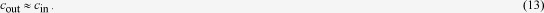


To achieve the condition in Eq. (12), we could set, for example, 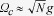, 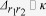 and 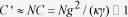. Therefore, assisted by Rydberg blockade interaction, our scheme can work in the single-atom weak-coupling regime, i.e., 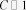, when the number of atoms 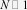.

Based on above analysis, when the photon pulse is reflected off the cavity, it achieves a conditional phase shift 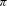, i.e., when the atoms are in state 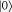, the photon experiences a phase shift 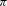, while there is no phase shift if the atoms are in state 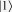. The physical understanding of these results can be seen from the so-called dark resonance^26^. As shown in Fig. 1(c), there are three resonant peaks for three-level cavity-EIT system. The central peak results from dark resonance^27^. When the atoms are in state 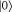, the Rydberg blockade interaction does not work (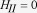). Thus the system of atoms and cavity mode is a typical three-level 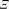-type system and its Hamiltonian 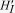 has a dark state


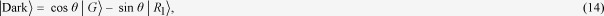


with 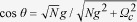 and 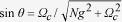. This dark state is decoupled from state 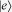 due to quantum interference in this three-level system. When the single photon is reflected off the cavity, the effect of dark resonance is equivalent to that of no atom coupled to the cavity^6^. Then the photon pulse will enter the cavity and leave it with a phase shift 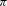. When the atoms are in state 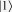, the Rydberg blockade interaction shifts the level 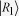 and moves the atomic system out of the dark state 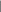
Dark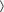. Therefore, the photon pulse, under certain conditions, will bounce back with no phase shift.

Now we describe in detail how to realize the photon-atom CPF gate. Initially, the atoms are prepared in an arbitrary superposition state of two logical states, i.e., 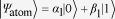, and the flying single-photon pulse is in superposition state of two orthogonal polarization components 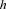 and 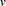, i.e., 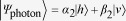. As shown in Fig. 1(a), the photon first passes a polarization beam splitter (PBS), which transmits only the 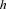 polarization component and reflects the v polarization component. Then the 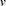 polarization component of the photon is reflected by the mirror 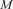 with nothing changed, while 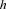 polarization component is reflected off the cavity and achieves a conditional phase shift 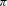. Thus the overall reflection from the cavity and the mirror 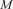 actually performs the CPF gate operation 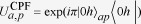 on atoms in cavity and the photon pulse, so that there is a phase shift 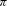 only when the atoms are in the state 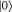 and the photon is in the polarization 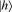.

**Figure 2:**
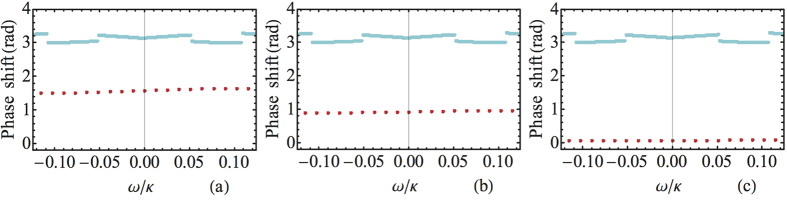
(Color online) The conditional phase shift vs the frequency of incoming photon pulse in units of 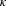, with the initial atomic states 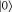 (solid curve) and 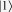 (dotted curve), for (**a**) 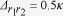, (**b**) 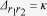 and (**c**) 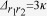. Other common parameters: 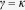, 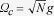, 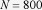, 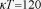, and the single-atom cooperativity 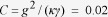.

We quantify the quality of the CPF gate between the flying optical photon and the Rydberg atomic ensemble through the numerical simulation. Following the method of Ref. [28], we perform numerical simulations with the assumption that the single-photon pulse is a Gaussian pulse, i.e., the pulse shape 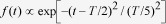, here 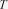 is the pulse duration. Our numerical simulations show that the conditional phase shift works well. First of all, the phase factor is approximately 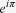 or 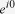 depending on the atomic state 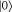 or 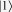 when 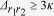, as depicted in Fig. 2. Note that there are some symmetrical phase jumps for the 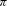 phase on both sides of center frequency, which was also observed in the single atom case^10^, however, the influence of these small phase jumps on the CPF gate is small, because most of the population of the photon pulse are around the center frequency when 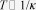. Second, this conditional phase factor is very insensitive to the variation of 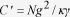. For instance, its variation is smaller than 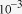 for 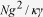 varying from 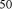 to 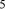, so that we do not need to know the exact number 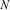 of the atoms in the optical cavity. Third, the phase shift has a high fidelity 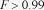 in the typical parameter region, i.e., 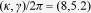 MHz Ref. [29] and 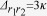 Ref. [20], on the assumption that 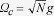, 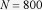, 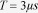 and the single-atom cooperativity 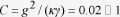.

**Figure 3:**
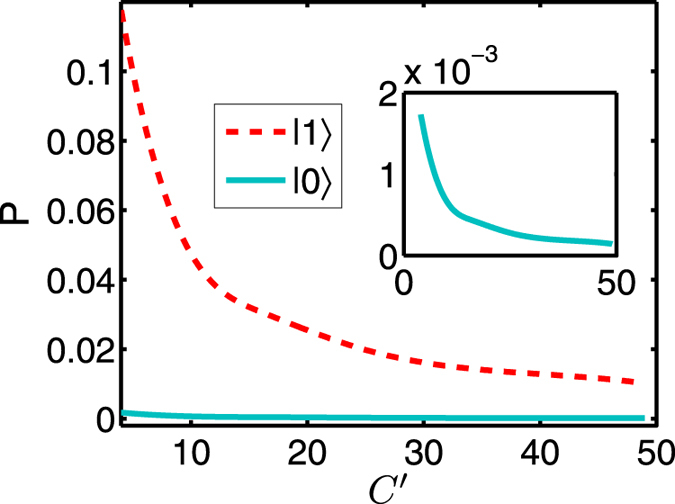
(Color online) The probability 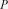 for the spontaneous emission loss as a function of 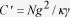, with the atomic states 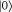 (solid curve) and 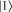 (dash curve). Other common parameters: 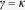, 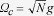, 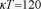, and 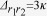.

Due to atomic spontaneous emission, the noise arises from photon loss which leads to a vacuum-state output. This noise yields a leakage error which means that the final state is outside of the qubit Hilbert space 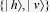. Figure 3 shows the probability 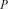 of spontaneous emission loss as a function of 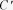 for the atomic states 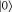 and 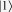. When the atoms are in state 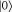, the numerical results show 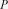 is smaller than 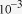. The physical reason for the results is that the dark state 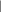
Dark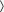 has no contribution from the excited state 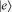 and the dark resonance process does not involve the state 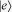. Since the population in state 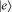 is zero, there is no spontaneous emission and hence no absorption. If the atoms are in state 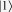, the curve is well simulated by the empirical formula 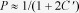. Other sources of photon loss come from the mirror scattering and absorption^16^,^17^,^18^. Note that these leakage errors only affect the probability to register a photon from each pulse and has no influence on the fidelity of its polarization state if a photon is registered for each qubit. So, the leakage errors induce small inefficiency of the CPF gate used for scalable quantum computation^8^,^9^.

## Discussion

Next we briefly give some discussion of our scheme. First, as shown in Fig. 2, there are some symmetrical phase jumps, which remain an open question. We will further study it in the future. Second, when the atoms are in state 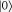, the photon can resonate to the cavity as it is under the 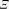-type cavity-EIT condition. Note that the cavity linewidth with this cavity-EIT dark resonance is reduced by a factor 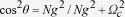 Ref. [30]. Therefore, the pulse duration 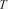 of the photons needs to satisfy the condition 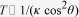. In our scheme, we assume 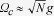, thus the pulse duration 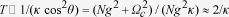.

Then we address the experiment feasibility of the proposed scheme. For a potential experimental system, we consider an optical cavity traps a ensemble of ultracold atoms within the volume 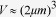 31,32. For the high 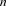-s (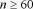) Rydberg states, one could achieve the strong blockade interaction with 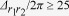 MHz and the small decay rate 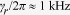 Ref. [20]. Typically, the relevant cavity parameters are 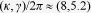 MHz Ref. [29] and thus 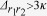. In the optical cavity, the cavity-atom coupling strength depends on the atomic position through the relation^28^


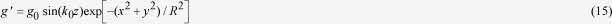


where 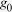 is the peak coupling strength in the antinodes, 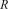 and 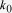 are, respectively, the waist and the wave vector of the Gaussian cavity mode, and 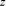 is assumed to be along the axis of the cavity. For the experimental realistic parameters of the cavity^29^, 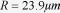, and 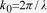, with 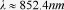 being the wavelength of cavity mode. Assume that the atomic number density of the atomic ensemble is 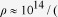
cm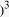 and thus about 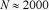 atoms within the volume 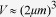 are coupled to the cavity mode with the collective cooperativity 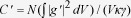 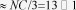, here 
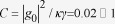
 is the peak cooperativity for a single atom coupled to the cavity.

In summary, we have proposed a scheme that realizes the CPF gate between a flying optical photon and an atomic ensemble. When a flying single-photon pulse is reflected off the cavity containing a Rydberg atomic ensemble, the dark resonance and Rydberg blockade induce a conditional phase shift 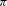, thus we can achieve the CPF gate between the photon and the atomic ensemble. Assisted by Rydberg blockade interaction, our scheme works in the 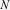-atoms strong-coupling regime, i.e., the collective cooperativity 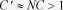. With a large number of atoms (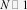), our scheme can work in the single-atom weak-coupling regime, i.e., 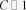, which significantly relaxes the requirement of the optical cavity for realization of the quantum CPF gate.

## Methods

Integrating Eq. (6) from an initial time 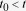 (the input) formally yields 25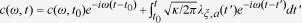, where 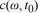 is the value of 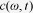 at 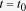. We assume that the frequency 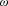 of input single-photon pulse is centered around the resonant frequency of the cavity mode and 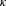 varies very slowly with the change of the frequency 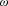 Ref. [25]. Then we substitute 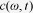 into Eq. (7) to get


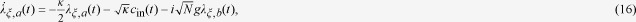


where we have used the relations 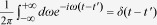 and 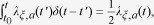 Where 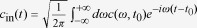 denotes the input field.

Taking the standard cavity input-output relation 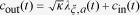 Ref. [25] and the adiabatic limit, i.e., setting the derivatives 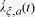, 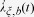 and 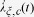 equal to zero, we obtain, from Eqs. (8,9) and (16),


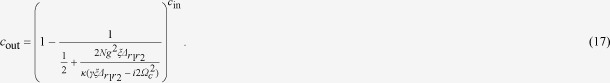


## Author Contributions

G.W.L. contributed the original concept of the theoretical model; Y.P.N. and S.Q.G. contributed to the development of the model; Y.M.H. performed the simulations and calculations; K.X. and X.M.L. contributed some idea to the model. Y.M.H., G.W.L., Y.P.N. and S.Q.G. discussed the results and wrote the manuscript.

## Additional Information

**How to cite this article**: Hao, Y. M. *et al.* Quantum controlled-phase-flip gate between a flying optical photon and a Rydberg atomic ensemble. *Sci. Rep.* **5**, 10005; doi: 10.1038/srep10005 (2015).
